# Evolution of the uniquely adaptable lentiviral envelope in a natural reservoir host

**DOI:** 10.1186/1742-4690-3-19

**Published:** 2006-03-20

**Authors:** LJ Demma, TH Vanderford, JM Logsdon, MB Feinberg, SI Staprans

**Affiliations:** 1 Program in Population Biology, Evolution and Ecology, and Emory Vaccine Center, Emory University, Atlanta, GA, USA; 2 Centers for Disease Control and Prevention, Division of Bacterial and Mycotic Diseases, 1600 Clifton Road, Mailstop D-63, Atlanta, GA 30333, USA; 3 Department of Biology, Emory University, Atlanta, GA. Current address: University of Iowa, Department of Biological Sciences, Roy J. Carver Center for Comparative Genomics, 301 Biology Building, Iowa City, IA 52242, USA; 4 Departments of Medicine and Microbiology and Immunology, and Emory Vaccine Center, Emory University School of Medicine, Atlanta, GA, USA; 5 Merck Vaccine Division, Merck and Company, Inc., 770 Sumneytown Pike, West Point, PA 19486, USA; 6 Emory Vaccine Center, 954 Gatewood Rd., Atlanta, GA, 30329, USA

## Abstract

**Background:**

The ability of emerging pathogens to infect new species is likely related to the diversity of pathogen variants present in existing reservoirs and their degree of genomic plasticity, which determines their ability to adapt to new environments. Certain simian immunodeficiency viruses (SIVcpz, SIVsm) have demonstrated tremendous success in infecting new species, including humans, resulting in the HIV-1 and HIV-2 epidemics. Although SIV diversification has been studied on a population level, the essential substrates for cross-species transmission, namely SIV sequence diversity and the types and extent of viral diversification present in individual reservoir animals have not been elucidated. To characterize this intra-host SIV diversity, we performed sequence analyses of clonal viral envelope (env) V1V2 and gag p27 variants present in individual SIVsm-infected sooty mangabeys over time.

**Results:**

SIVsm demonstrated extensive intra-animal V1V2 length variation and amino acid diversity (*le*38%), and continual variation in V1V2 N-linked glycosylation consensus sequence frequency and location. Positive selection was the predominant evolutionary force. Temporal sequence shifts suggested continual selection, likely due to evolving antibody responses. In contrast, gag p27 was predominantly under purifying selection. SIVsm V1V2 sequence diversification is at least as great as that in HIV-1 infected humans, indicating that extensive viral diversification in and of itself does not inevitably lead to AIDS.

**Conclusion:**

Positive diversifying selection in this natural reservoir host is the engine that has driven the evolution of the uniquely adaptable SIV/HIV envelope protein. These studies emphasize the importance of retroviral diversification within individual host reservoir animals as a critical substrate in facilitating cross-species transmission.

## Background

Most newly emerging human pathogens are zoonotic [1], yet little is known about the natural reservoirs from which these zoonoses emerge. RNA viruses, due to their extraordinary genomic variability, have been particularly capable of establishing infection in new host species [1-5]. As examples, the transfer of avian influenza A [6-8] and rodent hantavirus [9-12] from their natural reservoirs to create novel human outbreaks has been documented on several occasions [13,14]. Nonetheless, successful breaching of the host range barrier is relatively rare, with self-sustaining outbreaks in a new host species presumably requiring multiple mutational events. Two different simian immunodeficiency viruses (SIVs) from Central African chimpanzees and West African sooty mangabeys (SM) are inferred to have been transferred to humans by several independent zoonotic events, resulting in the introduction to humans of HIV-1 and HIV-2, respectively [15-18]. Although phylogenetic analyses of SIV sequences reveal considerable viral genetic diversity between different infected individuals [19], the magnitude of intra-animal viral diversity, the substrate for selection in cross-species transmission events, has not been studied. Furthermore, the mechanisms and tempo of the generation of viral variation in natural reservoir hosts are poorly understood.

Over 40 different species of African non-human primates harbor the CD4+ T cell tropic lentiviruses [20]. In these natural reservoir hosts, the SIVs do not cause AIDS, despite high viremia. Disease only develops upon transmission of SIV to new non-natural hosts such as humans or Asian macaques [21]. We have been studying the virologic and immunologic aspects of natural SIV infection in a colony of SIV-infected SMs at the Yerkes National Primate Research Center [22-24]. Although SIV-infected SMs are highly viremic, they manifest far lower levels of aberrant immune activation and apoptosis than are seen in pathogenic SIV and HIV infections and maintain preserved T lymphocyte populations and regenerative capacity [22,23]. Studies of the SIVsm viral variants obtained from different SMs demonstrate magnitudes of inter-animal viral diversity similar to that observed with different HIV-1 group M subtypes [19].

Variation in the viral surface proteins of zoonotic viruses is likely key to the ability of these agents to engage new host cell receptors and gain a foothold in new species. For influenza virus, amino acid changes and changes in glycosylation patterns in the viral hemagglutinin affect receptor binding specificity and host range [25,26]. For the SARS coronavirus (SARS-CoV) discreet variations in the spike protein are proposed to be important for viral tropism and animal-to-human transmission [27]. The HIV and SIV envelope (Env) proteins are extraordinarily genetically variable and highly glycosylated. HIV Env has evolved to tolerate considerable aa sequence flexibility, including variation in N-glyc sites, and to conformationally shield key receptor-binding domains [28]. This genetic and functional flexibility enables Env to escape from antibody responses and to utilize different co-receptors to gain efficient entry into target cells [29-35]. In our studies of the adaptation of SIVsm from a naturally infected SM to a new simian host (rhesus macaques) we observed that one of three phylogenetically distinct *env *variants could replicate to high levels in the newly infected macaques. These variants encoded a shorter variable region 1 loop and lacked two specific N-linked glycosylation sites (N-glyc sites) [24]. The pre-existence of viral *env *variants in naturally infected SMs that are capable of replicating to high levels in a new host species pointed to the importance of SIVsm diversity in the reservoir host in enabling cross-species transmission.

Studies of zoonotic RNA virus diversity have not focused on the variation that already exists in the source reservoir hosts; rather, the focus has largely been on the genetic variation and specific adaptive mutations that are observed in the newly emerged human pathogen [36,37]. While adaptive mutations are critical for efficient host-to-host propagation in the newly-infected species, viral diversity that is already extant in reservoir hosts is another important source of the genetic variation necessary for successful cross-species transmission. Here we describe extraordinarily high intra-host SIVsm *env *V1V2 diversity in naturally infected SMs, maintained by its high replication rate and positive selection most likely mediated by antibody responses. Ongoing evolution of an extremely mutable SIV *env *in the natural host explains the ease with which these lentiviruses can adapt to divergent host cellular environments and evade Ab responses in new host species.

## Results

### Magnitude of intra-host SIVsm diversity in naturally infected SMs

**Figure 1:**
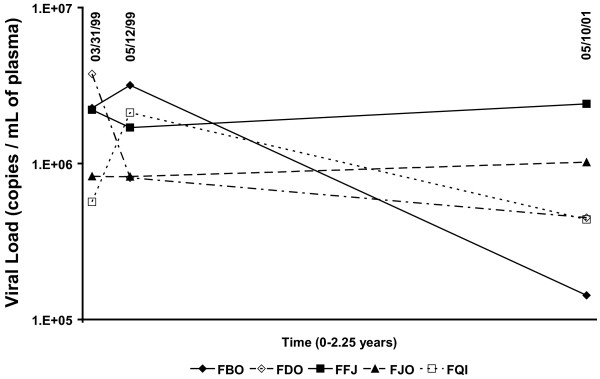
**Viral load quantification for five naturally infected sooty mangabeys**. Viral RNA in plasma obtained in 3/99, 5/99, and 5/01 was measured by a real-time RT-PCR assay designed to quantitatively detect the diverse SIVsm variants (viral RNA copies/mL).

Five naturally SIV-infected SMs (Table 1) were sampled three times over a 2-year period. Viral RNA in plasma obtained in 3/99, 5/99, and 5/01 was measured by a real-time RT-PCR assay designed to quantitatively detect the diverse SIVsm variants [23]. Time points were chosen so that evolution could be assessed over both shorter and longer time intervals. Viral load averaged 1.5 × 10^6 ^SIV RNA copies/ml plasma, and fluctuated modestly over the 2-year period (Figure 1). No clinical signs of AIDS were observed in any of the infected SMs over the study period.

**Table 1:** Summary of animals used in this study. Data was collected from five sooty mangabeys used in this study (housed at Yerkes Primate Research Center, Atlanta, GA).

**Animal Name**	**Virus subtype**	**Birthdate**	**Mean Viral Load (copies/mL)**	**No. V1V2 clones**	**No. *gag *clones**
**FFj**	1	04-20-88	2.11 × 10^6^	46	48
**FBo**	2	07-18-91	1.86 × 10^6^	73	23
**FDo**	3	07-29-91	1.67 × 10^6^	52	24
**FJo**	1	08-18-91	8.92 × 10^5^	58	32
**FQi**	1	05-20-87	1.04 × 10^6^	91	43

Multiple V1V2 *env *clones (range 15–29) and p27 *gag *clones (range 5–19) were sampled from each animal at each time point (Genbank Accession numbers AY733102-AY733566). *Env *and *gag *were chosen for analysis since they were thought to represent the extremes of diversity in SIV populations. These genes also differ in how the immune system detects them, with *env *V1V2 being exposed primarily to neutralizing antibodies [38] and *gag *p27 being recognized mostly through cellular immune responses [39]. The number of individual viral sequences analyzed (Table 1) combined with the sampling of variants over a short time interval (2 months) and a longer time interval (2 years) exceeds that reported in previous studies of SIV diversity in natural hosts [40-43].

**Figure 2:**
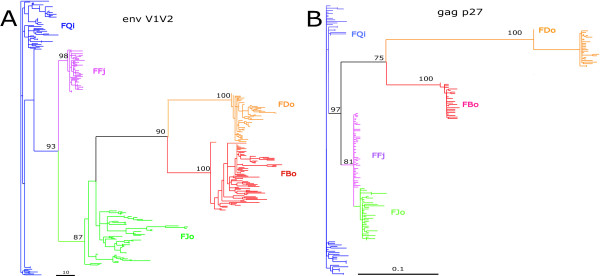
**Maximum likelihood trees of (A) all V1V2 variants, and (B) all *gag *variants**. A GTR model of evolution with empirically determined substitution rates was assumed. Bootstrap support is shown only for major lineages.

To characterize the overall evolutionary dynamics of natural SIV variation, we built maximum likelihood trees of both *env *V1V2 (Figure 2A) and *gag *p27 (Figure 2B) sequences. The SIVsm variants from each SM formed distinct clades in both genes, and the *env *and *gag *trees showed the same relationship between virus populations of the 5 animals. These results demonstrate that each host harbors a phylogenetically distinct population of SIVs, presumably as the result of infection with distinct viral populations and subsequent host-specific viral evolution.

**Figure 3:**
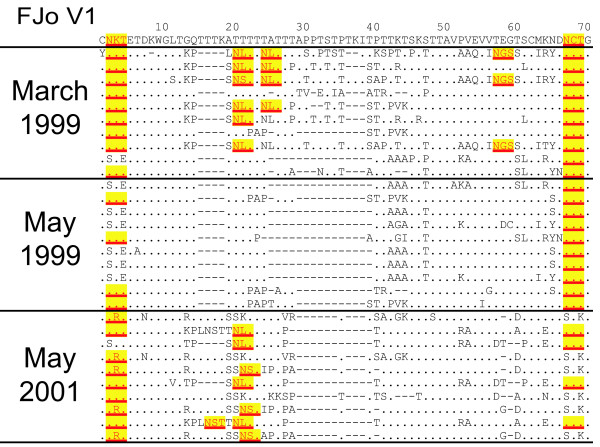
***Env *amino acid diversity of FJo SIVsmm sequences**. The consensus of all sequences is indicated at the top with the amino acid positions labeled above. Time points 1 (31-March-99), 2 (12-May-99) and 3 (10-May-01) are indicated by 1, 2, and 3 in the sequence titles. The glycosylation consensus motifs (NXT/S) are highlighted in yellow.

The translated *env *aa sequences (FJo, Figure 3; data from all animals can be obtained from THV) demonstrate significant V1V2 heterogeneity, including heterogeneity in numerous predicted N-glyc sites (NXS/T, where X can be any aa but proline). Considerable V1 length variations were observed (Table 2 and for example, Figure 3), such that alignment of this region required manual adjustment, and may not represent precise homology. There were no trends in V1V2 sequence length variation over time (data not shown). *Gag *aa alignments (available from THV) showed significantly less aa variation reflecting its highly conserved nature.

**Table 2:** Summary of intra-animal amino acid and nucleotide diversity and sequence length in V1V2 *env*. Pairwise distances were calculated using the Gamma distance method with gamma shape parameter of 0.3 in the program Mega 2.0 b. Shown are the mean, standard deviation, maximum, and minimum pairwise amino acid and nucleotide diversity and mean, maximum and minimum amino acid sequence length for each animal, at each time point.

**Animal**	**Date**	**Diversity (aa) **(Min, Max)	**St Dev (aa)**	**Diversity (nt) **(Min, Max)	**St Dev (nt)**	**Length (aa) **(Min, Max)	**# N-glyc sites **(Min, Max)
**FQi**	**3-99**	**0.097 **(0, 0.21)	**0.047**	**0.051 **(0, 0.115)	**0.024**	**142.11 **(140, 145)	**6.6 **(5, 9)
	**5-99**	**0.067 **(0, 0.13)	**0.037**	**0.033 **(0, 0.065)	**0.018**	**141.41 **(140, 144)	**7.1 **(5, 9)
	**5-01**	**0.087 **(0, 0.18)	**0.037**	**0.047 **(0, 0.107)	**0.017**	**142.64 **(140, 144)	**7.9 **(6, 9)
**FDo**	**3-99**	**0.041 **(0.01, 0.09)	**0.018**	**0.021 **(0.006, 0.042)	**0.009**	**143.77 **(142, 145)	**8.4 **(8, 9)
	**5-99**	**0.033 **(0, 0.07)	**0.014**	**0.020 **(0.003, 0.042)	**0.008**	**143.53 **(141, 145)	**8.5 **(7, 9)
	**5-01**	**0.059 **(0, 0.11)	**0.024**	**0.029 **(0.003, 0.059)	**0.011**	**144.89 **(141.149)	**7.5 **(4, 9)
**FJo**	**3-99**	**0.123 **(0.02, 0.26)	**0.052**	**0.063 **(0.006, 0.128)	**0.026**	**156.83 **(148, 163)	**7.9 **(6, 10)
	**5-99**	**0.076 **(0, 0.18)	**0.050**	**0.045 **(0, 0.113)	**0.028**	**148.43 **(145, 153)	**6.1 **(6, 7)
	**5-01**	**0.160 **(0, 0.38)	**0.082**	**0.088 **(0, 0.177)	**0.041**	**150.26 **(144, 152)	**6.1 **(3, 7)
**FBo**	**3-99**	**0.086 **(0, 0.2)	**0.041**	**0.045 **(0, 0.101)	**0.020**	**147.16 **(137, 156)	**6.6 **(5, 8)
	**5-99**	**0.118 **(0.01, 0.31)	**0.047**	**0.058 **(0.003, 0.136)	**0.022**	**146.64 **(137, 151)	**6.9 **(6, 8)
	**5-01**	**0.110 **(0, 0.21)	**0.047**	**0.053 **(0, 0.102)	**0.022**	**147.61 **(142, 153)	**6.6 **(6, 8)
**FFj**	**3-99**	**0.029 **(0, 0.08)	**0.015**	**0.017 **(0, 0.035)	**0.007**	**141.39 **(140, 145)	**7.8 **(7, 8)
	**5-99**	**0.051 **(0, 0.11)	**0.023**	**0.024 **(0, 0.046)	**0.010**	**141.42 **(133, 145)	**7.8 **(7, 8)
	**5-01**	**0.032 **(0, 0.07)	**0.018**	**0.016 **(0.006, 0.031)	**0.006**	**144.83 **(141, 149)	**7.6 **(6, 8)

Pairwise nt and aa diversity was calculated after removing regions of uncertain homology (gap-stripping) in V1, such that the values obtained for intra-host diversity represent minimum values. Average pairwise aa diversity was high in *env *V1V2 (average: 5.6%, range: 0 and 37.7%; Table 1) and low in *gag *p27 (average 1%; range: 0 and 7.1%, data not shown). The minimal diversity detected in *gag*, which was amplified under identical conditions, confirms that the observed V1V2 diversity is not the result of PCR-introduced mutation. In individual animals, the magnitude of nt and aa diversity did not change significantly over the 2-year observation period (Table 2). However, there appeared to be animal-to-animal variation in the extent of V1V2 diversity, with animals FFj and FDo exhibiting lower V1V2 nt and aa diversity than FJo and FBo (ANOVA p < 0.01, with Bonferroni adjustment). Nt and aa diversity were not correlated with viremia, suggesting that mechanisms other than or in addition to the magnitude of virus replication determine the extent of viral diversity. We cannot rule out that reduced diversity in FFj and FDo are the result of infection with less diverse virus populations.

### Positive selection maintains *env *V1V2 diversity

**Figure 4:**
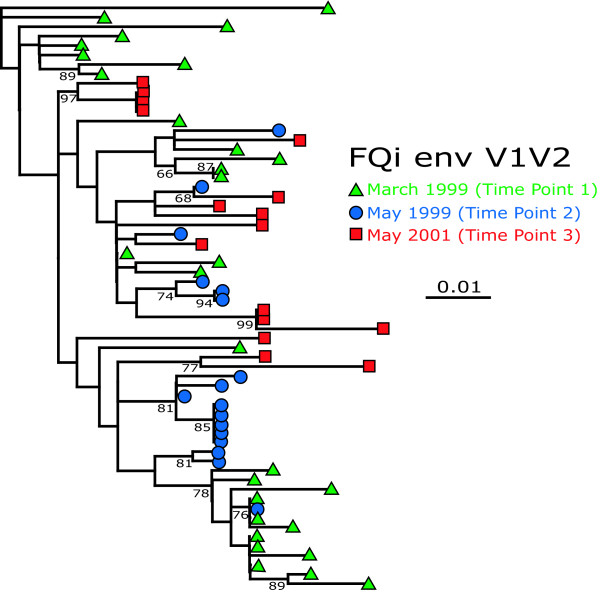
**Maximum likelihood tree of FQi**. V1V2 variants using the GTR+Γ+I model of substitution. >60% bootstrap support is indicated on the tree.

Although the magnitude of sequence diversity did not change over time, it was likely that *env *sequences at later time points had diverged from those sampled earlier. To investigate the temporal pattern of sequence evolution within each animal, all available samples from all three time-points for each animal were pooled and analyzed by maximum likelihood (Fig. 4; FQi). Sixteen of the nineteen (85%) bootstrap-supported clades from FQi contain variants from a single time point only. This pattern was repeatable amongst variants from all other animals; 100%, 80%, 69%, and 63% of bootstrap supported clades consisted of a single time point in animals FDo, FFj, FJo, and FBo, respectively. In an analysis of random trees, the number of matching time-point sequences that comprise a monophyletic group showed a Poisson distribution; 86% of variants did not form monophyletic clades with any other matching time-point variant (i.e., these sequences stood alone). Thus, the observed temporal clustering of SIVsm viral populations does not occur by chance alone (Kolmogorov-Smirnov test, p < 0.01).

**Figure 5:**
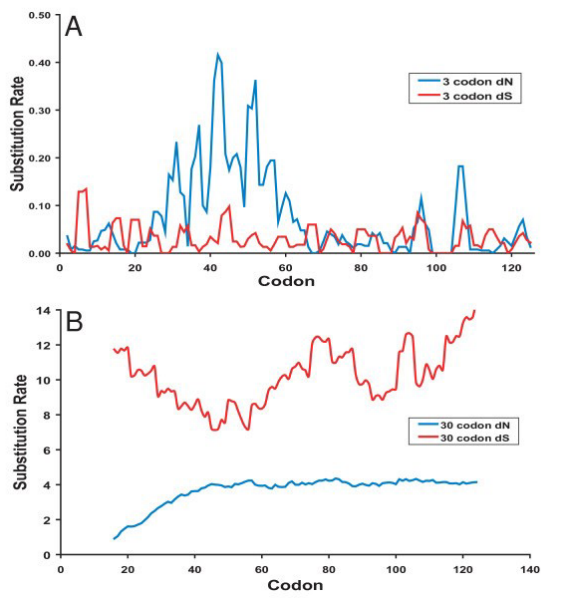
**Modes of selection in V1V2 and *gag***. (A) Positive selection (dN>dS) in *env *V1 and (B) purifying selection (dN<dS) in *gag p27 *within animal FQi is shown through a sliding window analysis of nonsynonymous and synonymous substitution rate.

Temporal phylogenetic structure in V1V2 suggested that continual V1V2 diversification was occurring. To look for evidence of positive selection, dN and dS were calculated at each site and averaged over a 3-codon sliding window for VIV2 (Fig. 5A) or 30-codon sliding window for p27 (Fig. 5B). These results confirmed that dN-dS>0 (p = 0.003, t-test) in V1 (aa's 25–55) in all animals, indicating positive selection. For p27, the same test showed that dS>dN along this gene (t-test, p < 0.001), indicating that purifying selection limits its diversity. V1 was consistently found to be under significant positive selection in all animals, except FFj (data not shown). By contrast, the few aa changes in p27 sequences in the different animals over time appeared random in nature except for a single partially fixed mutation in FDo.

### SIVsm *env *V1V2 sequences predict a highly glycosylated protein, with N-glyc site density being inversely correlated with Env diversification

Up to 10 N-glyc sites are contained within the SIVsm V1V2 regions sequenced in this study. In multiple locations overlapping consensus motifs (aa's 42–44, 52–54, and 95–107) are present, such that the exact site of glycosylation varies (Fig. 3). These overlapping consensus motifs are in particularly diverse regions of V1V2 and in regions of strong positive selection.

**Figure 6:**
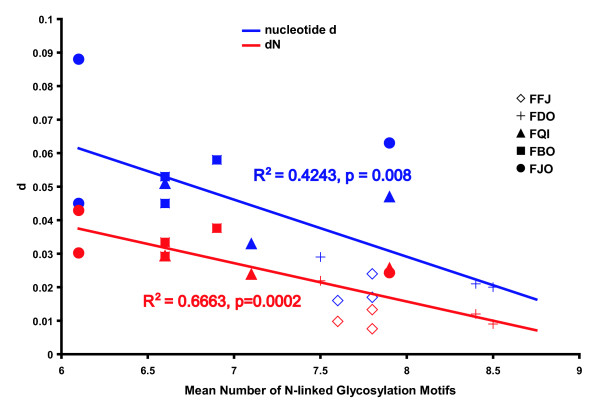
Glycosylation of SIVsmm V1V2 is inversely correlated with pairwise nucleotide diversity.

V1V2 clones from the five SMs contained variable numbers of N-glyc sites, ranging from 3 to 10. The average number of N-glyc sites among all animals was 7.2. There was no clear pattern of increased or decreased V1V2 *env *glycosylation with time. However, the mean number of N-glyc sites for FFj and FDo (7.8 and 8.2, respectively) was significantly higher than the other animals (average between 6.5 and 6.9; ANOVA, Tukey B, p < 0.001). An additional N-glyc site is found in V1 in the majority of sequences in FFj and FDo at position 45, but not in the other animals. There was also a smaller range of N-glyc sites per set of sequences in FFj and FDo (6–9) compared to other animals (3–10). As described, the FDo and FFj SIVsm populations were less diverse and had lower average dN compared to the virus populations found in the other 3 animals (Table 2). A significant inverse correlation between the mean number of N-glyc sites and both pairwise nt diversity and nonsynonymous substitutions was observed when combining data from all five SMs (p < 0.001, Fig. 6).

### Comparable levels of lentiviral env V1V2 diversification in SIVsm-infected natural hosts and HIV-infected humans

Diversification of the HIV genome in humans underlies its success in evading pharmacologic and immunologic selection pressures, and likely facilitates human-to-human transmission events. It has also been suggested that extensive virus diversification actually drives disease progression and the destruction of the immune system [44,45]. To compare the SIVsm genome diversity observed in natural hosts with that of HIV-1 in humans, longitudinally sampled *env *aa sequences from proviral DNA representing 9 untreated, chronically HIV-infected humans [46] were compared to our plasma RNA-derived SIVsm *env *data. Two time points were chosen from both the SM and the human dataset so that the interval between observations was approximately 2.5 years.

**Figure 7:**
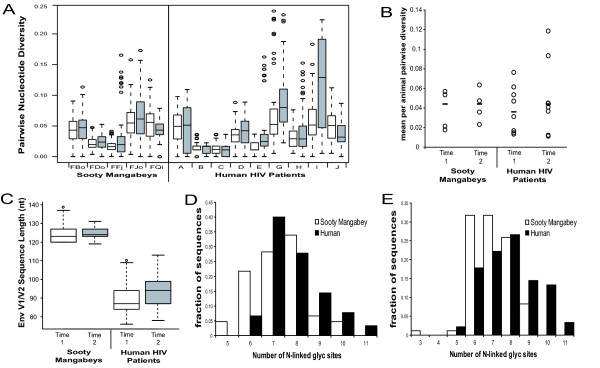
**Comparison of SIVsm and HIV-1 V1V2 sequences**. Longitudinal SIVsm and HIV-1 *env *sequences were aligned and homologous regions were compared with respect to nucleotide diversity, sequence length, and glycosylation density at an early time point (Time 1) and a time point approximately 2.5 years later (Time 2). (A) Standard box and whisker plots of the distributions of intra-animal pairwise nucleotide diversity. Time 1 is in white, time 2 is in gray, and circles represent outliers of the distribution. (B) Intra-animal average pairwise diversity at each time point. Median values are indicated with a slash. (C) Standard box and whisker plots of intra-animal *env *V1V2 sequence length at each time point. (D) and (E) N-linked glycosylation sites at time 1 and time 2, respectively. SMs are in white and humans are in black.

For the comparison of nucleotide sequence diversity, homologous regions surrounding V1V2 were aligned and gap-stripped. Average pairwise nucleotide diversity was calculated separately in each host at both time points (Figure 7A). Measures of SIVsm and HIV-1 nt diversity were not significantly different from each other within each time point (Figure 7B; p > 0.05, Mann-Whitney U test). Thus SIVsm V1V2 sequence diversity in the natural SM host is at least as great as, if not greater than that observed in HIV-1-infected humans, especially given that the archival nature of proviral sequences may overestimate the diversity of the actively replicating viral RNA population [47-49].

*Env *adapts not only through raw nt sequence variability, but also through variation in both sequence length and N-glyc site density and position. Substantial changes in these phenotypic parameters will affect the ability of *env *to utilize different co-receptors [50,51], evade neutralizing antibodies [52,53] and establish new infections in naïve hosts [54,55]. To elucidate differences in SIVsm and HIV-1 V1V2 sequence length and N-glyc site density variation, a pooled estimate of variance within each species was compared. Neither the variances of sequence length nor glycosylation density differed significantly between species at time point 1 although although humans had a greater variance in both parameters at time point 2 (F_max _test, p < 0.01). The variance of sequence length of SIVsm V1 decreased between the two time points (F_max _test, p < 0.005) suggesting that the magnitude of selection in SMs shifts over time, while in humans the variance remained stable (Figure 7C). The variation in glycosylation density (Figure 7D-E) remained relatively stable over time within both species except for a slight but non-significant expansion of variance in humans at time point 2.

## Discussion

To identify viral characteristics that may explain how the SIVs have successfully infected other primate species, we analyzed the types and extent of SIVsm diversification in naturally infected SMs. Our findings of high intra-host extremes of SIVsm V1V2 nt diversity extend previous studies of naturally SIV-infected SMs and African green monkeys (AGMs) [56-63] by demonstrating that viruses found within a single animal can vary by greater than 35% at the aa level. The ranges of aa diversity in some intra-host pairwise SIVsm V1V2 sequence comparisons in this study rival that of inter-animal comparisons [40]. As our diversity calculations exclude V1V2 length variation, they represent an underestimate of the true magnitude of viral diversity. V1V2 length polymorphisms would be predicted to have dramatic effects on SIVsm Env conformation and phenotypic diversity [64,65].

Positive selection in V1V2 appears to explain the observed *env *diversification. Specific sites in V1 were consistently selected for in four of the five animals. Our results agree with other studies of SIV and HIV selection, in which dN-dS was consistently greater than 0 [66-68]. However, the majority of previous studies of nonpathogenic SIV infection [56,69,70] calculated dN and dS by averaging over all sites, obscuring variation in selective pressure between aa sites. In addition to positive selection in V1V2, we detected temporal shifts in SIVsm populations, some of which involved the gain or loss of N-glyc sites.

Beyond aa sequence variation, the extensive glycosylation of the HIV and SIV envelope glycoprotein is thought to reduce protein epitope exposure and to facilitate viral evasion of antibody neutralization [28,52,53,55]. Ten potential N-glyc sites were recognized in the SIVsm V1V2 region, with the average virus encoding 7.2 N-glyc sites. The neutralization resistant SIVmac239 strain contains 8 predicted glycosylation sequences in the same region, while some other macaque-adapted SIVs appear to have fewer N-glyc sites, especially in the V1 region [28]. Thus, like SIVcpz in a naturally infected chimpanzee [71], SIVsm appears to be highly glycosylated in naturally infected SMs. Presumably, continually evolving antibody responses in these natural hosts maintain a highly glycosylated surface protein, albeit without effectively suppressing virus replication. Our observation of an inverse relationship between N-glyc site density and SIVsm V1V2 sequence diversity might result from the more highly glycosylated viral variants being better shielded from the diversifying selection pressures of anti-SIV antibodies than less glycosylated variants, as recently suggested for HIV [55]. Thus, antibody-mediated pressures on the SIVsm envelope glycoprotein appear to exist in this natural host reservoir species, and serve to continually select for adaptations in envelope sequence and structure.

In contrast to *env*, SIVsm *gag *p27 was under strong purifying selection in infected SMs. Temporal analyses of *gag *p27 demonstrated no evidence of the fixation of specific aa substitutions, suggesting that *gag *p27 is not the target of strong selective pressures such as those that might be expected if anti-Gag cellular immune responses were present. These observations corroborate our findings that natural SM hosts mount limited cellular immune responses to SIV infection [22,23,72].

Comparison of our SIVsm plasma RNA-derived V1V2 sequences and a set of HIV-1 envelope sequences obtained from proviral DNA [46], while not the ideal comparison, demonstrates that natural SIVsm V1V2 diversity is as great, if not greater than that observed in HIV-1-infected humans. Since average pairwise diversity is an indirect measure of viral effective population size [73], these results suggest that an equivalent number of target cells are infected in both SM and human immunodeficiency virus infections. The similar levels of viral variation may also indicate that selective forces acting on *env *V1V2 are comparable in both SIVsm-infected natural mangabey reservoir hosts and in HIV-infected humans. A caveat of these SIV and HIV sequence comparisons is that this protein is quite divergent between the two viruses, and it is possible that this region of *env *could be under different functional and immune selection pressures in the two hosts.

As V1V2 is primarily a target of the antibody response, it will be important to more thoroughly characterize in natural hosts SIVsm variation in viral genome regions known to encode multiple cytotoxic T lymphocyte (CTL) epitopes in non-natural hosts (such as humans and macaques). Such studies could help to elucidate the selective pressures exerted by the natural host on other genome regions and inform us as to the potential for genetic plasticity in viral genes that are targeted by current CTL-focused HIV vaccine strategies.

The observation that high-level virus replication and extensive sequence diversification do not harm SMs is consistent with the notion that the direct effects of SIV replication are not sufficient to explain AIDS [44,45,74]. Instead, our studies of natural host responses to infection indicate that indirect mechanisms, such as host inflammatory immune responses elicited by virus infection, likely play a role in the development of AIDS in new non-natural hosts [22,23]. Because the humoral immune responses in naturally infected SMs do not significantly suppress virus replication, they may actually serve to promote the continuous selection of *env *sequences and structures [75]. This helps to explain how the unique SIV/HIV Env structure has evolved in lower primates, resulting in a virus that is extremely difficult to neutralize [75,76]. This continuous diversifying selection pressure likely also serves to generate variants with expanded cell tropisms that are well suited to adapt to new host cellular environments [24]. For instance, a spectrum of variant SIV Env conformations with differing requirements for the levels of CD4 on target cells might help to breach species differences in CD4 molecules, which are generally not as well conserved as the viral co-receptors such as CCR5 [77,78]. Thus, high viral variability and recombination within a natural reservoir host or host population will increase the likelihood that variants with the ability to replicate in new host species exist. The ongoing intra-host diversification of human-adapted RNA viruses, such as HIV and hepatitis C virus, enables these viruses to continually respond to changing pressures, such as those imposed by immune responses and antiviral therapies, making treatment of these human diseases a formidable challenge [52,79,80].

## Conclusion

The extent of intra-host SIVsm *env *diversification in its natural reservoir likely underlies the ease with which certain SIVs infect new host species [20,24]. As new human pathogens emerge, much focus is placed on viral evolution in the newly infected hosts, such as adaptive mutations that facilitate robust replication and pathogenesis. However, our studies of SIVsm demonstrate that an important source of viral variation and thus adaptive potential can be found within the viral populations of individual reservoir host animals. This extensive intra-animal viral variation, which is likely key to facilitating cross-species transmission events, may be a common zoonotic signature among diverse emergent pathogens.

## Materials and methods

### Specimens and RT-PCR

Five age-matched, naturally SIV-infected SMs from the colony at the Yerkes National Primate Research Center, Atlanta, GA were chosen for study. Individual animals were between 8 and 12 years of age and were estimated to have been infected for approximately 3 to 9 years, based on available HIV-2 seroconversion data. Thus, all animals were born in, and acquired their SIVsmm infection in, captivity. Group housing of the animals confounds identification of potential donor-recipient pairs. Plasma from animals FQi, FJo, FFj, FDo, and FBo was obtained on 3-13-99, 5-12-99, and 5-10-01 and viral RNA was extracted and quantified using a real-time RT-PCR assay designed to quantitatively detect the diverse SIVsmm variants [23]. Viral RNA was diluted such that approximately 2500 copies of viral RNA were used in a Superscript™ First-Strand Synthesis System for RT-PCR (Invitrogen Corporation, Carlsbad, CA.), following the protocol provided, primed by random hexamers. 2 μL of cDNA from the RT-PCR was used for PCR amplification of both *env *V1V2 and *gag *p27 with Qiagen HotStar Taq (Qiagen Inc., Chatsworth, CA.). The *env *V1V2 region was amplified with the forward primer V1V2DF (5'-TTTGATGCNTGGAAYAAYAC-3') corresponding to bp 6774–6792 of the SIVsmmH4 genome (GenBank accession no. X14307), and the reverse primer V1V2DR (5'-CATAGCATCCCARTARTGCTT-3') corresponding to bp 7217–7238 of the SIVsmmH4 genome. The primer pair amplified a 421 bp fragment spanning the V1–V2 hypervariable region of envelope. The *gag *region was amplified using shortgagF1 (5'TTAAGTCCAAGAACATTAAATGC-3') and shortgagR (5'GTAGAACCTGTCTACATAGCT-3') which correspond to bp 1493–1515 and 19371957 of SIVsmmH4, respectively, yielding a 421 bp product of the 5' end of the p27 capsid protein. Primers were designed by choosing highly conserved regions from an alignment of all SIV and HIV2 *env *and *gag *sequences from the HIV sequence database [81]. Conditions for each reaction were 30 min. at 50°C, 15 min. at 95°C, followed by 40 cycles of 94°C for 1 min., 52°C for 30 s, and 72°C for 1 min. A final extension time was carried out for 5 min. at 72°C. No-template controls and negative controls from the RNA extraction were used in each set of reactions, both RT and PCR, to ensure that no cross contamination occurred at either step. RT-PCR sensitivity was determined to be = 500 copies per reaction.

### Cloning and DNA sequencing

PCR products from each sample were run on a 1.5% low-melt agarose gel. The resulting 425 bp V1V2 or 421 bp *gag *product was extracted and cloned into the pCR4-TOPO vector (TOPO TA Cloning Kit, Invitrogen). From Rodrigo et al. [82] it was determined that if 2500 copies of viral RNA are used in the RT-PCR reaction, 20 clones picked from the PCR product will be unique. Therefore, approximately 20 clones from V1V2 and 10 from *gag *(due to lower expected diversity in this conserved gene) at each time point and each animal were randomly selected and sequenced using the M13F and M13R primers using the dye terminator cycle sequencing method with an MJ Research automated sequencer.

### Sequence and phylogenetic analyses

Sequences were aligned using the program CLUSTAL X [83], followed by manual adjustment using MacClade 4.0 [84] and BioEdit Sequence Alignment Editor [85]. Non-aligned regions of length variation in V1 and V2 were removed (corresponding to nucleotides 6932–6974), and sequences containing internal stop codons or frame shifts were also excluded from analysis as these are thought to be PCR artifacts [86].

For tree construction, the Modeltest program [87] was used to construct and evaluate the DNA substitution models used. Based on the Modeltest results phylogenetic analysis on sequences obtained from successive time points during the acute infection was performed by maximum likelihood (ML) using the program Treefinder [88]. The general-time-reversible model, which allows for rate variation between sites [89-91], was used, and the shape parameter (α) of the gamma distribution used in this model was estimated, as were base frequencies and substitution rate parameters. Bootstrap support was determined with 1,000 resamplings of the ML tree using distance methods in PAUP4.0b10*, incorporating the estimated rate parameters. Phylogenetic trees were constructed from all clones obtained from V1V2 and *gag *and also separately on V1V2 and *gag *sequences obtained from each animal at each time point by maximum likelihood (ML) using the program Treefinder.

The cumulative number of nonsynonymous (dN) and synonymous (dS) nucleotide substitutions was estimated using **SNAP**, Synonymous/Non-synonymous Analysis [81] which calculates rates of nucleotide substitution based on the method of Nei and Gojobori [92], and incorporating a statistic developed in Ota and Nei [93]. Viral diversity at each time point was determined by calculating the pairwise nucleotide distances for each of the clones using the method of Tamura and Nei [94], and pairwise amino acid distances using the Gamma distance method in the program MEGA 2.1 [95]. Average dN and dS were calculated using the modified Nei-Gojobori method in MEGA 2.1. Phylogenetic trees constructed with synonymous or nonsynonymous sites only were constructed in MEGA 2.1 using distance methods, incorporating the Tamura-Nei model of nucleotide substitution with gamma-distributed rates. All statistics were computed using SYSTAT 10.

### Temporal analysis of V1V2 sequences from individual animals

In order to show that viral populations do not vary randomly through time, random trees of all variants from each animal were generated and the number of matching time-point sequences that formed a monophyletic clade was counted for each random tree. For the random trees, the number of matching time-point sequences that comprise a monophyletic group are Poisson distributed. The Kolmogorov-Smirnov test was used to compare our observed trees with those built from randomly sampled sequences.

### Comparison of SIVsm and HIV-1 diversity

*Env *nt sequences from 9 patients of a study of 10 HIV-infected patients [46] were compared to our SIVsm *env *data with respect to nt diversity, sequence length variation, and predicted N-linked glycosylation site diversity. For V1V2 nt diversity comparisons, sequences from both SMs and patients were aligned and stripped of gaps. Pair-wise estimates of intra-host nt diversity were calculated using Mega 2.1 [96]. For sequence length variation, alignments (including gaps) of both SIVsm and HIV-1 were pared down to the V1V2 region as defined by the flanking regions of extreme conservation. For this test, homology of each amino acid site was not as important as the overall homology of the region. Mean-squared error variance was determined by ANOVA in R [97] for both glycosylation density and sequence length in each species at each time point. Variances were compared manually using an F_max _test.

### Data deposition footnote

Genbank Accession Nos: AY733102-AY733566

## Abbreviations

SIV, simian immunodeficiency virus; SM, sooty mangabey; RM, rhesus macaque; nt, nucleotide; aa, amino acid; Ab, antibody; NAb, neutralizing antibody.

## Competing interests

The author(s) declare that they have no competing interests.

## Authors' contributions

LJD, MBF, and SIS conceived and designed the experiments. LJD carried out the reverse transcription, PCR, and cloning. JML contributed reagents and manpower for sequencing. LJD, THV, and JML conceived and performed statistical and phylogenetic analyses of the sequence data. LJD, THV, and SIS wrote the manuscript. All authors read and approved the final manuscript.
